# Synthesis of *L*-Ornithine- and *L*-Glutamine-Linked PLGAs as Biodegradable Polymers

**DOI:** 10.3390/polym15193998

**Published:** 2023-10-05

**Authors:** Gülce Taşkor Önel

**Affiliations:** Department of Analytical Chemistry, Faculty of Pharmacy, Erzincan Binali Yıldırım University, Yalnızbağ, Erzincan 24002, Türkiye; gulce.onel@erzincan.edu.tr

**Keywords:** poly(lactic-*co*-glycolic acid), polyester, *L*-ornithine, *L*-glutamine, amino acid, ROP, EDC coupling, biosimilars, in vitro degradation

## Abstract

*L*-ornithine and *L*-glutamine are amino acids used for ammonia and nitrogen transport in the human body. Novel biodegradable synthetic poly(lactic-*co*-glycolic acid) derivatives were synthesized via conjugation with *L*-ornithine or *L*-glutamine, which were selected due to their biological importance. *L*-ornithine or *L*-glutamine was integrated into a PLGA polymer with EDC coupling reactions as a structure developer after the synthesis of PLGA via the polycondensation and ring-opening polymerization of lactide and glycolide. The chemical, thermal, and degradation property–structure relationships of PLGA, PLGA-*L*-ornithine, and PLGA-*L*-glutamine were identified. The conjugation between PLGA and the amino acid was confirmed through observation of an increase in the number of carbonyl carbons in the range of 170–160 ppm in the ^13^C NMR spectrum and the signal of the amide carbonyl vibration at about 1698 cm^−1^ in the FTIR spectrum. The developed PLGA-*L*-ornithine and PLGA-*L*-glutamine derivatives were thermally stable and energetic materials. In addition, PLGA-*L*-ornithine and PLGA-*L*-glutamine, with their unique hydrophilic properties, had faster degradation times than PLGA in terms of surface-type erosion, which covers their requirements. *L*-ornithine- and *L*-glutamine-linked PLGAs are potential candidates for development into biodegradable PLGA-derived biopolymers that can be used as raw materials for biomaterials.

## 1. Introduction

Biodegradable polymers have many developing different applications in the medical field [1,2,3,4]. In recent years, research on environmentally friendly polymeric designs for biomaterials has become increasingly widespread around the world [5,6,7,8]. For instance, it has been reported that an *L*-lysine-loaded poly(lactic-*co*-glycolic acid) (PLGA) microparticle system, which supports cell proliferation and angiogenesis, has been developed as a nontoxic, biocompatible, and cost-effective microparticle system for rapid vascularization [9]. Therefore, a biomaterial with antibacterial properties that accelerates wound healing with good cell harmony has been developed [10,11]. A good example is also reported in biocompatible conductive and porous PLGA scaffolds, which exhibit stretchable neural or cardiac tissue regeneration functions due to electrical conductivity to improve proper cell function [12]. Poly(lactic acid) (PLA) and poly(glycolic acid) (PGA) are 100% biodegradable, nontoxic, and ecofriendly biopolymers [13,14]. However, the fact that they are expensive polymers that degrade relatively quickly and have low melting points [15] prevents the widespread use of PLA [16] and PGA [17].

PLGA, obtained through the ring-opening polymerization (ROP) of lactide and glycolide, is a polymer approved by the US Food and Drug Administration (FDA) that can be used in clinical applications in humans [18]. Studies on the regulation of degradation time that consider molecular weight, polydispersity (Đ), crystallinity, tacticity, pH, biological conditions, hydrophilic and hydrophobic functional groups, and stereo sequence in new-generation polymers have been remarkable for PLGA [19]. A useful approach for the design of new biodegradable polymers was reported to support alkaline hydrolysis in both crystalline and amorphous regions of polyesters, while enzymatic degradation was observed in amorphous regions [20]. Since PLGA contains only ester bonds, the soft segment undergoes degradation and the hard segment degrades much more easily than that of polymers [21]. For this reason, the design of polyurethane-like hard segment-degradable polymers containing amide bonds could result in the acceleration of the degradation time [4]. Derivative polymers based on PLGA with amino acids are also a good alternative that can reduce the degradation time.

*L*-glutamine is a unique molecule classified as a semiessential or conditionally essential amino acid with two nitrogen side chains. The amino acid *L*-glutamine is used for ammonia and nitrogen transport in the human body [22]. *L*-ornithine is also an amino acid produced by the separation of urea from *L*-arginine in the body’s urea cycle. It is an important biochemical reagent involved in the excretion of excess nitrogen from the body [23]. PLGA derivatives created as biomaterials via the conjugation of amino acids and PLGA are expected to have excellent biocompatibility and regulated biodegradable properties with more hydrophilicity compared with PLGA [24].

Peptide-mimicking polymers, which have been intensively developed in recent years, also offer remarkable results. Vandermeulen et al. designed a PEG-*b*-peptide hybrid block copolymer correlating with their self-assembly behavior and proposed it for DNA complexation [25]. Poly(*N*-methacryloyl-L-leucine) was designed as a peptide-mimetic coating material in the research by Raczkowska et al. and reported to exhibit high protein adsorption and cell adhesion over wide temperature and pH ranges [26]. The review by Hancock et al. highlights the benefits and limitations of peptide-mimetic antimicrobial polymeric materials. Peptide-mimicking polymers containing host defense peptides containing cationic groups, e.g., lysine, have been reported to have better activity than their tertiary and quaternary ammonium counterparts regarding bacterial cell surface binding and membrane-disrupting abilities [27]. Thus, peptide-mimetic polymers rich in *L*-ornithine and *L*-glutamine with a primary amine functional group are likely to have antibacterial properties.

For new-generation biomaterials, amino acid-linked PLGA systems are ideal for micro/nanoparticle drug carriers [28,29]. In addition, PLGA structures supported by amino acids have been shown to meet the requirements of tissue or bone regeneration [30]. As an effective vaccine candidate, Margoroni et al. developed a peptide-mimicking polymeric nanoparticle system from a short tumor necrosis factor α (TNFα)-peptide and PLGA. The peptide-mimicking nanoparticles were taken up by fully functionalized dendritic cells, presented *Leishmania* antigen, and induced the development of T-cell priming [31]. The PLGA-PEG grafted antimicrobial peptide nanoparticle system developed by Ramoa et al. for the treatment of wound infections is the subject of a study that provides a different perspective on peptide polymer structures. Promising progress was reported with the formulation prepared via grafting an antimicrobial peptide onto PLGA-based nanoparticles, which resulted in wound infections healing in between 15 min and 1 h [32]. In summary, the polarity of the structure increases with the conjugation of amino acids to PLGA in the polyester structure. In another summary, the capacity to make H-bonds increased. In this way, the novel PLGA derivatives could demonstrate a positive improvement in biological interaction.

This research aims to design and synthesize novel amino acid-linked PLGA derivatives as biosimilars. In this study, PLGA was first synthesized from lactide and glycolide with ring-opening polymerization. In a second step, the synthesis was easily completed with the EDC coupling reaction of PLGA and amino acid. Detailed chemical identifications of the amino acid-linked PLGA derivatives were performed using ^1^H and ^13^C NMR spectroscopy and attenuated total reflectance (ATR)–Fourier transform infrared (FTIR) spectrometry. The molecular weights of the samples were also determined with gel permeation chromatography (GPC), the thermal transitions of the polymers were examined with differential scanning calorimetry (DSC) and thermogravimetric analysis (TGA), and the hydrophilicity and surface-free energies of the polymers were evaluated with contact angle measurements. Also, in vitro degradation studies were performed for the novel polymers and the degradation images were observed via scanning electron microscopy (SEM).

## 2. Materials and Methods

### 2.1. General

All reagents were of commercial quality and reagent grade solvents were used without further purification. 3,6-Dimethyl-1,4-dioxane-2,5-dione (99%) (CAS number 95-96-5), 1,4-dioxane-2,5-dione (≥99%) (CAS number 502-97-6), and 1-dodecanol for synthesis (CAS number 112-53-8) were obtained from Sigma-Aldrich Co. (St. Louis, MO, USA). Stannous 2-ethylhexanoate (95%) (CAS number 301-10-0) was purchased from Abcr GmbH Co. (Karlsruhe, Germany). 1-Ethyl-3-(3-dimethylaminopropyl)carbodiimide-hydrochloride (EDC-HCl) (≥99%) (CAS number 25952-53-8) was obtained from Carl Roth GmbH+ Co. (Karlsruhe, Germany). The solvents, dichloromethane (DCM) (CAS number 75-09-2) and methanol (MeOH) (CAS number 67-56-1), were supplied by Sigma-Aldrich Co. Before use, DCM was stored in the presence of a 4 Å molecular sieve.

Chemical identifications of PLGAs were carried out in terms of an explanation of the NMR and IR spectra, and determination of molecular weight, thermal properties, and morphologic properties. ^1^H NMR and ^13^C NMR spectra were followed on a Bruker Avance 300 MHz spectroscopy (Bruker Daltonics, Billerica, MA, USA), and reported in CDCl_3_. ^1^H NMR and ^13^C NMR chemical shifts (*δ*) were reported in parts per million relative to either tetramethylsilane (TMS) (*δ* = 0 ppm) as an internal standard or the residual solvent peak as follows: CDCl_3_ = 7.26 (^1^H NMR), CDCl_3_ = 77.16 (^13^C NMR). IR spectra were acquired in reflectance mode using a Nicolet 6700 FT-IR spectrometer from Thermo Fisher Scientific (Waltham, MA, USA), using the ATR accessory. The spectral range was 4000–400 cm^−1^; each spectrum was the result of 16 scans. DSC experiments were recorded on a Hitachi Exstar X-DSC7000 from SII NanoTechnology Inc. (Tokyo, Japan). Samples were scanned over a temperature range of 25–400 °C and at a heating rate of 10 °C min^−1^ under the N_2_ flow. Samples of a mass of 5 mg were used. Thermal stability was measured using a Hitachi STA 7300 TG/DTG/DTA from SII NanoTechnology Inc. under a heating rate of 20 °C min^−1^ from 100 to 800 °C and a nitrogen flow rate of 40 mL min^−1^. The molecular weight determinations of PLGA and the derivatives were studied using gel permeation chromatography (GPC) at METU PAL Laboratories (Ankara, Türkiye). GPC data were obtained using a Malvern OmniSEC instrument (Malvern Panalytical, Malvern, UK), equipped with a refractive index detector. Tetrahydrofuran was used as the solvent. Samples were passed through the defensive column (Phenogel—7.8 mm × 50 mm) and two columns (Phenogel—7.8 mm × 300 mm) at 0.9 mL/min. The column furnace temperature was fixed at 35 °C. Molecular mass distributions were calculated relative to narrow polystyrene reference standards. The surface hydrophilicity and free energies of the PLGA derivatives were measured with water contact angles by using an optical tensiometer (attention, Theta Lite, Stockholm, Sweden). The contact angle measurements were performed at least 10 times for each sample. The test liquids, water, formamide (HCONH_2_), and diiodomethane, were used with bromonaphthalene. The total surface-free energies (SFEs) and polar and dispersive components were calculated using the Owens, Wendt, Rabel, and Kaelble (OWRK) method. The morphologies were determined by using an FEI Quanta Feg 450 SEM microscope (Hillsboro, OR, USA). A thin layer of Au was used to cover the samples via sputter coating.

### 2.2. Synthesis of poly(lactic-co-glycolic Acid) (PLGA)

PLGA was synthesized using the ROP method and polycondensation reaction with a ratio of lactide [L]: glycolide [G] of 75:25, as in my previous studies [33]. Since PLGA was resynthesized, the identification of the chemistry of PLGA is repeated.

PLGA: white solid, ^1^H NMR (300 MHz, CDCl_3_) δ_H_ 5.23–5.15 (–CH–), 4.91–4.60 (–CH_2_–), 1.56 (–CH_3_), ^13^C NMR (125 MHz, CDCl_3_) δ_C_ 175.13 (CO), 169.38 (CO), 71.46 (–CH–), 60.78 (–CH_2_–), 16.75 (–CH_3_), FT-IR (neat, cm^−1^) 3003.58 (υCH_3_), 2925.08 (υCH_3_), 1750.72 (υC=O), 1445.06 (δCH_2_), 1385.24 (δCH_3_), 1249.21 (τCH_2_), 1087.55 (υC–O–C), GPC (THF, RI): M_n_ = 3000 g/mol, Đ = 3.438.

### 2.3. Synthesis of PLGA-L-Orn and PLGA-L-Gln

**Scheme 1:**
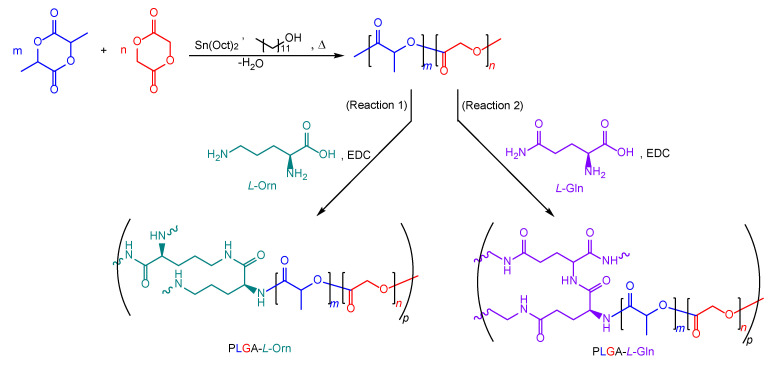
Synthesis steps of PLGA, PLGA-L-Orn (Reaction 1), and PLGA-L-Gln (Reaction 2).

The derivative polymer structures were synthesized from the PLGA and amino acids with the formation of an amide bond, in the presence of EDC [34]. L-Orn (Reaction 1) and L-Gln (Reaction 2) were selected separately as amino acids to react with PLGA (Scheme 1). In the first step of the EDC conjugation reaction, PLGA (100 mg) and EDC (48 mg) were added to a round-bottom flask equipped with a magnetic stirrer in anhydrous DCM (15 mL). Then, the amino acid (10 mg) was added to the reaction medium and continually stirred at room temperature for 48 h. The solvent was evaporated with a rotary evaporator and the crude PLGA was precipitated with cold MeOH. The creamy-white solid was washed with an ice-cold mixture of Et_2_O, MeOH, and dH_2_O (3 × 10 mL) to remove any residual chemicals and dried under a high vacuum. The product was stored at 8 °C.

PLGA-L-Orn: white solid, ^1^H NMR (300 MHz, CDCl_3_) δ_H_ 8.0, 6.5–6.0 (–NH–), 5.28–5.12 (lactidyl–CH–), 5.05–4.69 (glycolidyl–CH_2_–), 4.36 (L–Orn–CH–), 3.76–3.05, 1.60–1.35 (L–Orn–CH_2_–), 1.50 (–CH_3_), ^13^C NMR (125 MHz, CDCl_3_) δ_C_ 175.17 (CO), 174.07 (CO), 159.90 (CO), 71.32 (–CH–), 66.43 (–CH_2_–), 60.72 (–CH–), 37.18, 37.02, 35.61, 25.26, 23.51 (–CH_2_–), 20.01 (–CH_3_), FT-IR (neat, cm^−1^) 3295.69 (NH), 2982.41, 2925.59 (υCH), 1742.05 (υC=O), 1698.47 (υC=O), 1445.27 (δCH_2_), 1384.91 (δCH_3_), 1347.25 (τCH_2_), 1188.30 (υ=C–O), 1087.44 (υC–O–C), GPC (THF, RI): M_n_ = 12,300 g/mol, Đ = 1.975.

PLGA-L-Gln: white solid, ^1^H NMR (300 MHz, CDCl_3_) δ_H_ 8.1, 6.6–5.9 (–NH–), 5.29–5.10 (lactidyl–CH–), 5.08–4.62 (glycolidyl–CH_2_–), 4.37 (L–Gln–CH–), 3.78–3.07, 1.69–1.32 (L–Gln–CH_2_–), 1.49 (–CH_3_), ^13^C NMR (125 MHz, CDCl_3_) δ_C_ 175.32 (CO), 166.70, 166.23 (CO), 159.87 (CO), 71.91 (–CH–), 66.52 (–CH_2_–), 60.40 (–CH–), 37.41, 37.25, 35.74, 35.70, 25.81, 23.26 (–CH_2_–), 20.12 (–CH_3_), FT-IR (neat, cm^−1^) 3299.32 (NH), 2971.75, 2922.19 (υCH), 1742.16 (υC=O), 1698.47 (υC=O), 1446.78 (δCH_2_), 1385.12 (δCH_3_), 1350.63 (τCH_2_), 1178.70 (υ=C–O), 1087.33 (υC–O–C), GPC (THF, RI): M_n_ = 21,400 g/mol, Đ = 2.565.

### 2.4. In Vitro Degradation Studies

To determine the hydrolytic degradation of the PLGA derivatives, the samples were prepared as equal circular discs (radius = 0.5 cm) and kept in a phosphate-buffered saline solution, PBS (containing 120 mM of NaCl, 2.7 mM of KCl, and 10 mM of phosphate salts, with a final pH of 7.4) (0.1% *w*/*v*), at 37 °C. The degradation percentages of the polymers were calculated from the weight loss percentage of the PLGA derivatives, determined gravimetrically (number of samples, *n* = 5). The samples were weighed (*W*_0_) before incubation. Then, samples were removed weekly, rinsed three times with water, lyophilized, and weighed (*W*_1_) [4]. The degradation percentage was calculated from the weight loss percentage using the following Equation (1):

(1) $$\%\;\mathrm{Weight}\;\mathrm{loss}=\frac{W_{0}-W_{1}}{W_{0}}\times 100$$

In addition, the degradation morphologies of the polymers were visualized via scanning electron microscopy (SEM, FEI Quanta Feg 450, Hillsboro, OR, USA).

## 3. Results and Discussion

### 3.1. ROP of Lactide and Glycolide

In this study, PLGA was synthesized via the ROP method from lactide and glycolide monomers in a ratio of 75:25, respectively (Scheme 1). The reason for choosing the L:G/75:25 ratio was to study the polymer that optimized properties such as polymer structure, molecular weight, end group, crystallinity, and glass transition temperature [35]. Stannous octoate was used as a catalyst and 1-dodecanol as an initiator in the synthesis of the block copolymers.

**Figure 1:**
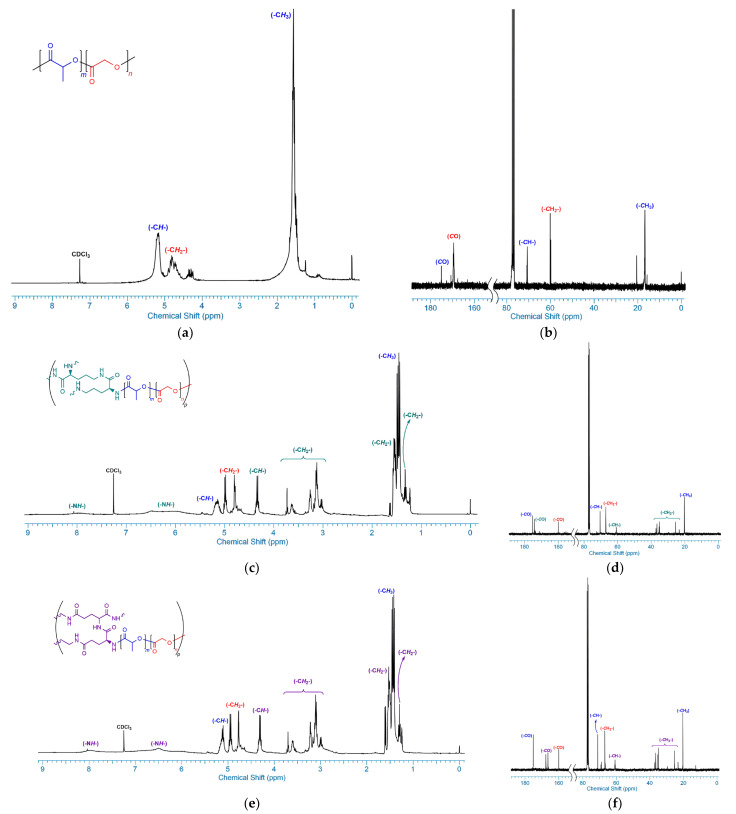
^1^H NMR spectra of (**a**) PLGA, (**c**) PLGA-*L*-Orn, and (**e**) PLGA-*L*-Gln and ^13^C NMR spectra of (**b**) PLGA, (**d**) PLGA-*L*-Orn, and (**f**) PLGA-*L*-Gln.

The ^1^H and ^13^C NMR spectra of PLGA in deuterated chloroform are shown in detail in Figure 1a,b. In Figure 1a, the –C*H*– proton belonging to the lactidyl chain is signaled at *δ* 5.24–5.14 ppm, and the –C*H*_3_ protons are also signaled at *δ* 1.55 ppm, while the –C*H*_2_– protons belonging to the glycolidyl chain is signaled at *δ* 4.92–4.61 ppm, as reported in the literature [36]. In addition, the characteristic carbonyl carbon peaks of the lactidyl and glycolidyl chains, shown at *δ* 175.13 ppm and *δ* 169.38 ppm, respectively, in Figure 1b, supported the structure of the PLGA.

**Figure 2:**
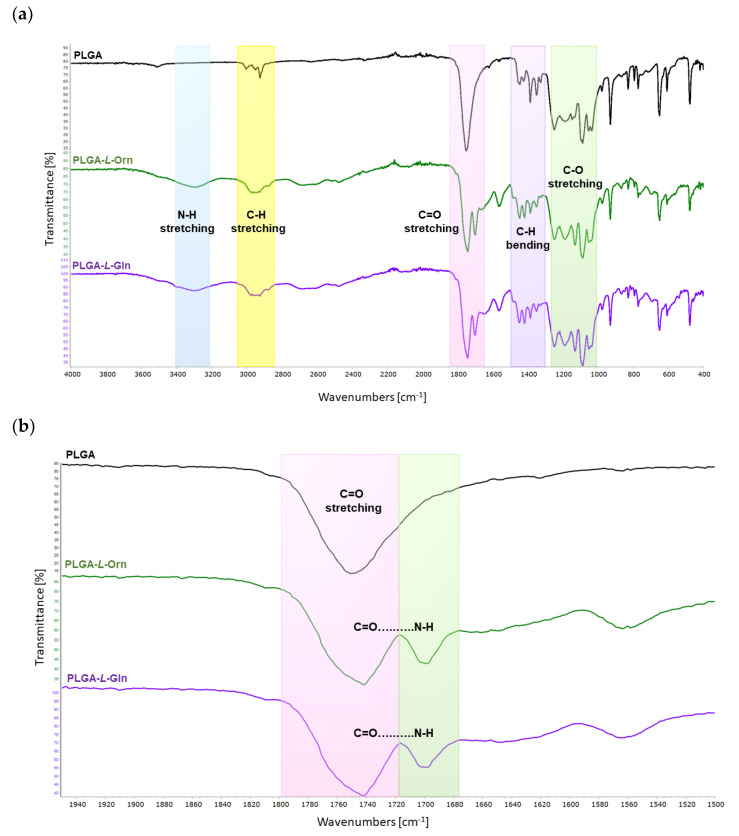
FTIR-ATR spectra of PLGA, PLGA-*L*-Orn, and PLGA-*L*-Gln; (**a**) 4000–400 cm^−1^ regions and (**b**) N-H and C=O vibration stretching regions.

In the FTIR spectrum of the PLGA, the signals at 3003–2925 cm^−1^ corresponded to typical C–H stretching. Typical C=O stretching bands of PLGA were observed at 1750 cm^−1^. Some low-density signals between 1445 and 1325 cm^−1^ were shown to be the C–H bending bands of PLGA. In addition, characteristic C–O stretching bands of PLGA were observed at 1249–1035 cm^−1^, as shown in Figure 2.

### 3.2. EDC Coupling of PLGA and L-ornithine (PLGA-L-Orn)

The PLGA was conjugated to *L*-Orn through the EDC coupling reaction. 1-Ethyl-3-(3-dimethylaminopropyl)carbodiimide (EDC) is the most ideal reagent for coupling reactions of carboxylic acid and amino functional groups due to its high water solubility [37]. The EDC coupling reaction was completed within 48 h as specified in the method description. The reaction mixture had a homogeneous transparent white appearance. The highly viscous liquid crude product was solidified by drying in a vacuum and cooling.

The ^1^H and ^13^C NMR spectra confirmed the chemical structure of the synthesized PLGA-*L*-Orn, which is shown in detail in Figure 1c,d. The broad singlets in the range of nearly *δ* 8.0 and *δ* 6.5–6.0 ppm observed in Figure 1c represent amide NH signals belonging to the *L*-Orn units. In Figure 1c, the quartet signal at *δ* 4.36 ppm belonging to the -CH- protons and various multiple or triplet signals between *δ* 3.76–3.05 and *δ* 1.60–1.35 ppm belonging to the methylene protons confirm the chemical structure accuracy of the *L*-Orn moiety of PLGA. Also, the carbonyl peak of *L*-Orn was observed at *δ* 159.90 ppm, while the signal belonging to the lactidyl chains was observed at *δ* 175.17 ppm and the signal belonging to glycolidyl chains was observed at *δ* 174.07 ppm, as shown in Figure 1d.

In the FTIR spectrum of the PLGA-*L*-Orn, the important peak at 3295 cm^−1^ corresponded to N-H stretching bands of the amide bond between *L*-Orn and PLGA. The signals at 2982–2925 cm^−1^ exhibited typical C-H stretching vibrations. Typical C=O stretching bands of PLGA were observed at 1742 cm^−1^. Another important peak at 1698 cm^−1^ corresponded to C=O…N-H carbonyl stretching vibrations belonging to the amide bonds of PLGA-*L*-Orn. Some low-density signals between 1445 and 1347 cm^−1^ were detected as C-H bending bands of PLGA. In addition, characteristic C-O stretching bands of PLGA were observed at 1248–1041 cm^−1^, as shown in Figure 2.

### 3.3. EDC Coupling of PLGA and L-glutamine (PLGA-L-Gln)

The PLGA was conjugated to *L*-Gln through the EDC coupling reaction. At the end of the 48 h reaction, a homogeneous transparent white high-viscosity liquid was also obtained for PLGA-*L*-Gln. The pure PLGA-*L*-Gln product was prepared using the same purification methods as those used for PLGA-*L*-Orn and stored at 8 °C.

The ^1^H and ^13^C NMR spectra exhibited the chemical structure of the synthesized PLGA-*L*-Gln, which can be observed in detail in Figure 1e,f. The chemical structure of PLGA-*L*-Gln was very similar to that of PLGA-*L*-Orn, as can be observed in the NMR spectra. The excess carbonyl group of the *L*-glutamine chains of the PLGA structure at *δ* 166.70 and *δ* 166.23 ppm can be observed in Figure 1f. According to Figure 1e, the broad signals around *δ* 8.1 and *δ* 6.6–5.9 ppm corresponded to the amide N*H* protons of the *L*-Gln units of the PLGA.

In the FTIR spectrum of the PLGA-*L*-Gln, the important peak at 3299 cm^−1^ corresponded to N-H stretching bands of the amide bond between *L*-Gln and PLGA. The signals at 2971–2922 cm^−1^ exhibited typical C-H stretching vibrations. Typical C=O stretching bands of PLGA were observed at 1742 cm^−1^. Another important peak at 1698 cm^−1^ corresponded to C=O…N-H carbonyl stretching vibrations belonging to the amide bonds of PLGA-*L*-Gln. Some low-density signals between 1446 and 1350 cm^−1^ were detected as C-H bending bands of PLGA. In addition, characteristic C-O stretching bands of PLGA were observed at 1248–1047 cm^−1^, as shown in Figure 2.

### 3.4. Thermal Properties of PLGA, PLGA-L-Orn, and PLGA-L-Gln

The thermal properties of the PLGA, PLGA-*L*-Orn, and PLGA-*L*-Gln were determined using thermogravimetric analysis (TGA) and differential scanning calorimetry (DSC) (Figure S8 in the Supplementary Materials).

Thomas et al. formulated that the glass transition temperature (*T*_g_) has a positive correlation with the number average molecular weight of the polymer [38]. PLGA, PLGA-*L*-Orn, and PLGA-*L*-Gln were observed to have *T*_g_ values of 26.5, 46.1, and 43.2, respectively. The *T*_g_ results were influenced by molecular weight, monomer ratio, end group, and increased crystallinity in PLGA-*L*-Orn and PLGA-*L*-Gln compared with PLGA. The increase in H-bonding capacity, increase in molecular weight, and end group differentiation may have contributed to the increase in the *T*_g_ of PLGA-*L*-Orn and PLGA-*L*-Gln derivatized from low-molecular-weight PLGA [4].

**Figure 3:**
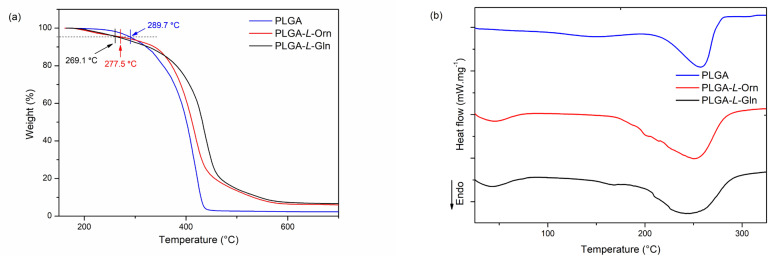
TGA (**a**) and DSC (**b**) curves of PLGA, PLGA-*L*-Orn, and PLGA-*L*-Gln.

DSC was used to study the thermal transition of the polymers. The DSC analysis results for a sequence with PLGA, PLGA-*L*-Orn, and PLGA-*L*-Gln exhibited typical endothermic peaks with melting at 256.9, 251.8, and 244.7 °C, respectively (Figure 3b and Table 1). Furthermore, a melting temperature was observed for all polymers, indicating the presence of crystalline domains in the PLGA, PLGA-*L*-Orn, and PLGA-*L*-Gln chains.

The decomposition temperature of the polymers was characterized by a heating rate of 20 °C min^−1^ under a nitrogen flow. The decomposition temperatures (*T*_d_) of PLGA, PLGA-*L*-Orn, and PLGA-*L*-Gln at 5% mass loss were 289.7, 277.5, and 269.1 °C, respectively. The decomposition temperature of PLGA is higher than those of PLGA-*L*-Orn and PLGA-*L*-Gln. In this case, since the thermal decomposition temperatures of PLGA-*L*-Orn and PLGA-*L*-Gln were reduced compared with that of PLGA, their thermal stability decreased. Thermal degradation temperatures of amino acids vary between 185 and 280 °C [39]. The reason for the decrease in stability could be due to the fact that *L*-Orn and *L*-Gln, which are rich in heteroatoms, differentiate the polarity of PLGA. In addition, it could be caused by the differences in the monomer binding sites of the PLGA derivatives and the crystallinity differentiation in the chemical structures. The structural stability of the PLGA, PLGA-*L*-Orn, and PLGA-*L*-Gln could also be demonstrated from the TG curves. As shown in Figure 3a, the thermal decomposition temperature decreased slightly with amino acid derivatization but indicated good thermal stability. Therefore, the addition of an amino acid segment to PLGA could favor stability [40].

The enthalpy of fusion (Δ*H*) was calculated from the areas of the endothermic peaks. The Δ*H* values of PLGA, PLGA-*L*-Orn, and PLGA-*L*-Gln were 127, 172, and 178 J g^−1^, respectively. PLGA-*L*-Orn and PLGA-*L*-Gln required much more energy than PLGA due to the presence of an amide bond in their chemical structures and their higher molecular weights [41].

### 3.5. Molecular Weight Analysis

The molecular weights of PLGA, PLGA-*L*-Orn, and PLGA-*L*-Gln are shown in Table 2 and were analyzed with GPC (Figure S9 in the Supplementary Materials).

The PLGA had a number average molecular weight (*M_n_*) of 3.000 g mol^−1^ and a weight average molecular weight (*M_w_*) of 10.400 g mol^−1^ with a polydispersity index (Đ) of 3.438 according to the results of the GPC analysis (Table 2).

The PLGA-*L*-Orn had a number average molecular weight (*M_n_*) of 12.300 g mol^−1^ and a weight average molecular weight (*M_w_*) of 24.300 g mol^−1^ with a polydispersity index (Đ) of 1.975. The PLGA-*L*-Gln had a number average molecular weight (*M_n_*) of 21.400 g mol^−1^ and a weight average molecular weight (*M_w_*) of 54.900 g mol^−1^ with a polydispersity index (Đ) of 2.565. The weight average molecular weights of the PLGA derivatives were found to be higher than the number average molecular weights, resulting in higher Đ values. Since the Đ value is the ratio of *M_w_*/*M_n_*, it is an important parameter for obtaining information about the chain configuration such as whether it is a linear, branched, chain length, crosslinked, or network formation. The high Đ values of the PLGA derivatives indicate that the chemical structures are far from monodispersity. Due to the amino functional groups at both ends of *L*-Orn and *L*-Gln, PLGA derivatives might be expected to promote crosslinking or branching.

### 3.6. Evaluation of Hydrophilicity of PLGA, PLGA-L-Orn, and PLGA-L-Gln

The hydrophilicity and surface-free energies (SFEs) of the PLGA derivatives were determined to clarify the surface erosion rates (Table 3).

The water contact angle values of PLGA, PLGA-*L*-Orn, and PLGA-*L*-Gln were 89.4, 58.6, and 52.7°, respectively. PLGA-*L*-Orn and PLGA-*L*-Gln showed similar water contact angle results. However, the results supported the increase in hydrophilicity. In addition, the biomaterials with water contact angle values less than 60° were reported to have good cell attachment behavior [42]. In addition, the publication by Gazvoda and Vukomanovic reported that hydrophilicity leads to surface erosion [43]. The results showed that the wettability of PLGA-*L*-Orn and PLGA-*L*-Gln was improved.

The lower contact angle values also reflect an increased surface energy [44]. More energetic PLGAs were designed with the presence of amino acids. The total SFEs were found to be 38.2, 53.7, and 58.0 mN/m for PLGA, PLGA-*L*-Orn, and PLGA-*L*-Gln, respectively. The polar component of the SFE increased with the rise in the number of surface treatments of the polymers, whereas the polar components of the PLGA-*L*-Orn (9.8 mN/m) and PLGA-*L*-Gln (10.3 mN/m) were found to increase compared with that of the PLGA (4.0 mN/m). The results exhibited that PLGA derivatives that are hydrolytic polymers with many more polar regions are more susceptible to hydrolytic degradation.

In addition, in accordance with the results reported for temperature-sensitive peptide-mimetic polymer coatings, the wettability values investigated through water contact angle measurements supported an increase in hydrophilicity and increases in protein adsorption and cell adhesion [26]. Thus, it is envisaged that the synthesized PLGA-amino acid derivatives may be candidates for biocompatible surfaces.

### 3.7. In Vitro Degradation Properties of PLGA, PLGA-L-Orn, and PLGA-L-Gln

The degradation rates and mechanisms of PLGA derivatives depend on their monomer sequence, stereochemical architecture, molecular weight, Đ value, crystallinity, morphology, tacticity, hydrophilic or hydrophobic functional groups, and susceptibility under physiological conditions [4]. The biodegradation properties of the PLGA, PLGA-*L*-Orn, and PLGA-*L*-Gln films were investigated in hydrolytic media at 37 °C for 100 days.

**Figure 4:**
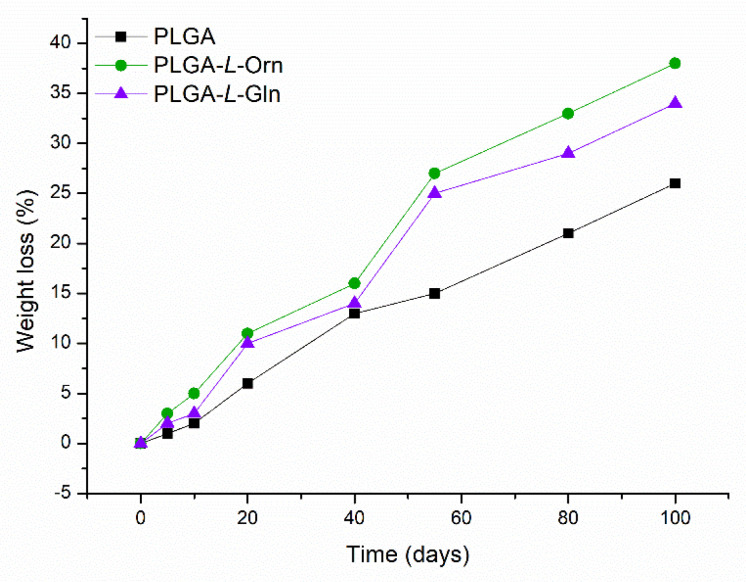
Biodegradation profiles of PLGA, PLGA-*L*-Orn, and PLGA-*L*-Gln in hydrolytic medium (PBS, 0.1 M, pH 7.4) at 37 °C for 100 days.

The degradation performance of PLGA, PLGA-*L*-Orn, and PLGA-*L*-Gln is presented in Figure 4 as weight loss percentage. The PLGA films degraded by 25% wt. in PBS media after 100 days. The 100-day degradation rate for PLGA-*L*-Orn was 38% wt. in PBS media, while it was 34% for PLGA-*L*-Gln. The degradation rates of the amino acid-derived PLGAs were increased and improved compared with that of PLGA. This may be due to the conjugation of hydrophilic groups on *L*-Orn and *L*-Gln with the PLGA structure and increased chemical interaction [45]. Similar results have been reported in the literature for faster degradation of amino acid-conjugated polymers [46,47].

**Figure 5:**
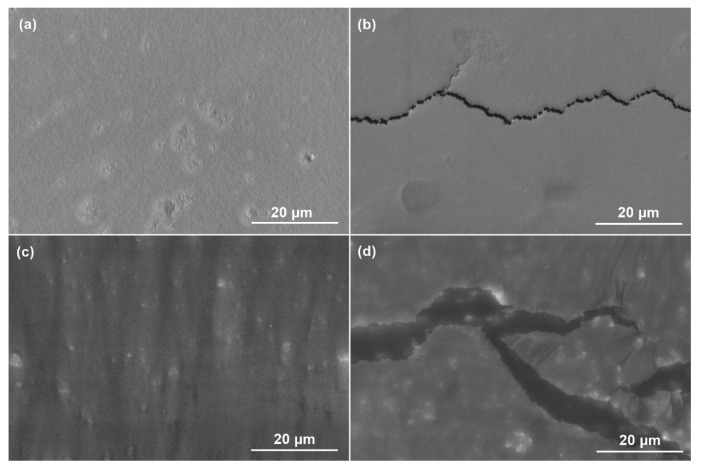
SEM images showing the in vitro hydrolytic degradation morphologies of (**a**) PLGA-*L*-Orn, (**c**) PLGA-*L*-Gln on the 1st day; and (**b**) PLGA-*L*-Orn, (**d**) PLGA-*L*-Gln on the 25th day.

It is known that there are two types of polymer degradation processes: bulk erosion and surface erosion [48]. The 25-day degradation morphologies of PLGA-*L*-Orn and PLGA-*L*-Gln can be observed in Figure 5. The smooth and regular surfaces are shown for PLGA-*L*-Orn in Figure 5a and for PLGA-*L*-Gln in Figure 5c. In Figure 5b,d, the fractures show that surface erosion started for PLGA-*L*-Orn and PLGA-*L*-Gln under hydrolytic degradation conditions. The reason for why amino acid-derived PLGAs were prone to surface erosion was the acceleration of the degradation process due to the presence of hydrophilic groups. The fact that linear mass loss was observed is one of the results supporting surface erosion. Multilayered irritated PLGA films were reported by Joachim Loo et al. to be ideal polymers for controlled drug-delivery systems due to their preference for degradation by surface erosion [49]. In controlled drug-delivery systems, polymers exhibit ideal behavior, with mass loss from the surface while maintaining their geometry. However, in the presence of enzymatic interactions or physiological conditions with acidic/basic pH variables, surface-type erosion could be converted to bulk erosion [50]. The results of this research show that the biodegradation properties of PLGA-*L*-Orn and PLGA-*L*-Gln with surface-type erosion make them suitable for biomaterial applications such as controlled drug release systems. However, the amino acid derivative structures of PLGA also have the potential to change the degradation mechanism from surface-type erosion to bulk-type erosion under certain physiological conditions or enzymatic interactions.

## 4. Conclusions

*L*-ornithine- and *L*-glutamine-linked PLGAs were successfully synthesized via EDC coupling reactions for the first time. The ^1^H and ^13^C NMR spectra proved the existence of a covalent bond between the amino acid and the PLGA. In addition, an amide bond, indicating the formation of the PLGA-amino acid conjugation, was displayed using FTIR analysis. The results showed that the conjugation of *L*-ornithine and *L*-glutamine to PLGA resulted in a thermally stable and energetic material. According to the water contact angle results of PLGAs derivatized with amino acids, it was observed that their hydrophilic properties increased compared with those of PLGA. Furthermore, the more hydrophilic structures of PLGA-*L*-Orn and PLGA-*L*-Gln showed faster degradation times than that of PLGA with surface-type erosion, which meets the requirements for biomaterials. Biodegradable *L*-ornithine- and *L*-glutamine-linked PLGAs are potential candidates for development as faster-degrading PLGA-derived biopolymers for biomaterials such as drug-delivery systems. Overall, this work provides important guidance for future applications of PLGAs derivatized with amino acids.

## Figures and Tables

**Table 1:** Thermal properties of PLGA, PLGA-*L*-Orn, and PLGA-*L*-Gln.

Sample	*T*_g_ (°C) ^a^	*T*_m_ (°C) ^b^	*T*_d_ (°C) ^c^	Δ*H* (J g^−1^) ^d^
PLGA	26.5	256.9	289.7	127
PLGA-*L*-Orn	46.1	251.8	277.5	172
PLGA-*L*-Gln	43.2	244.7	269.1	178

^a^ *T*_g_ is the glass transition temperature determined with the heating trace of DSC. ^b^ *T*_m_ is the melting temperature determined with the heating trace of DSC. ^c^ *T*_d_ is the decomposition temperature at 5% weight loss determined with TGA. ^d^ Δ*H* is the enthalpy of fusion determined with DSC.

**Table 2:** Molecular weight properties of PLGA, PLGA-*L*-Gln, and PLGA-*L*-Orn.

Sample	$\bar{M_{n}}$ (g/mol)	$\bar{M_{w}}$ (g/mol)	Đ
PLGA	3000	10,400	3.438
PLGA-*L*-Orn	12,300	24,300	1.975
PLGA-*L*-Gln	21,400	54,900	2.565

**Table 3:** Water Contact Angles and SFE Values for the PLGA, PLGA-*L*-Gln, and PLGA-*L*-Orn.

Sample	Water Contact Angle (°)	Total SFE: γ_*s*_ (mN/m)	Dispersive Component of SFE: $\gamma_{s}^{d}$ (mN/m)	Polar Component of SFE: $\gamma_{s}^{p}$ (mN/m)
PLGA	89.4 ± 2.0	38.2	36.6	4.0
PLGA-*L*-Orn	58.6 ± 1.4	53.7	35.1	9.8
PLGA-*L*-Gln	52.7 ± 2.1	58.0	34.9	10.3

## Data Availability

Data are contained within the article and Supplementary Materials.

## Supplementary Materials

The following supporting information can be downloaded at https://www.mdpi.com/article/10.3390/polym15193998/s1: Figure S1: ^1^H NMR spectrum of PLGA; Figure S2: ^13^C NMR spectrum of PLGA; Figure S3: ^1^H NMR spectrum of PLGA-*L*-Orn; Figure S4: ^13^C NMR spectrum of PLGA-*L*-Orn; Figure S5: ^1^H NMR spectrum of PLGA-*L*-Gln; Figure S6: ^13^C NMR spectrum of PLGA-*L*-Gln; Figure S7: FTIR spectra of PLGA, PLGA-*L*-Orn, and PLGA-*L*-Gln; Figure S8: TGA (a) and DSC (b) curves of PLGA, PLGA-*L*-Orn, and PLGA-*L*-Gln; Figure S9: GPC measurements of PLGA*-L*-Orn and PLGA-*L*-Gln.
