# Supplementary material for: Synthesis of L-Ornithine- and L-Glutamine-Linked PLGAs as Biodegradable Polymers
Source: Polymers (Basel). 2023 Oct 5;15(19):3998. doi: 10.3390/polym15193998 (PMC10575337; doi:10.3390/polym15193998)
Supplement: Supplementary file 1 [file polymers-15-03998-s001.zip › polymers-2598668-supplementary.pdf]

Supplementary Materials

Synthesis of *L*-ornithine- and *L*-glutamine-Linked PLGAs as Biodegradable Polymers

Gülce Taşkor Önel

Department of Analytical Chemistry, Faculty of Pharmacy, Erzincan Binali Yıldırım University, Yalnızbağ, Erzincan 24002, Türkiye; gulce.onel@erzincan.edu.tr

1. ^{1}H and ^{13}C NMR spectra of PLGA, PLGA-*L*-Orn, PLGA-*L*-Gln

2. FTIR spectrum of PLGA, PLGA-*L*-Orn, PLGA-*L*-Gln

3. TGA and DSC Thermograms of PLGA, PLGA-*L*-Orn, PLGA-*L*-Gln

4. Gel permeation chromatography measurement of PLGA-*L*-Orn and PLGA-*L*-Gln

1. ^{1}H and ^{13}C NMR spectra of PLGA, PLGA-*L*-Orn, PLGA-*L*-Gln

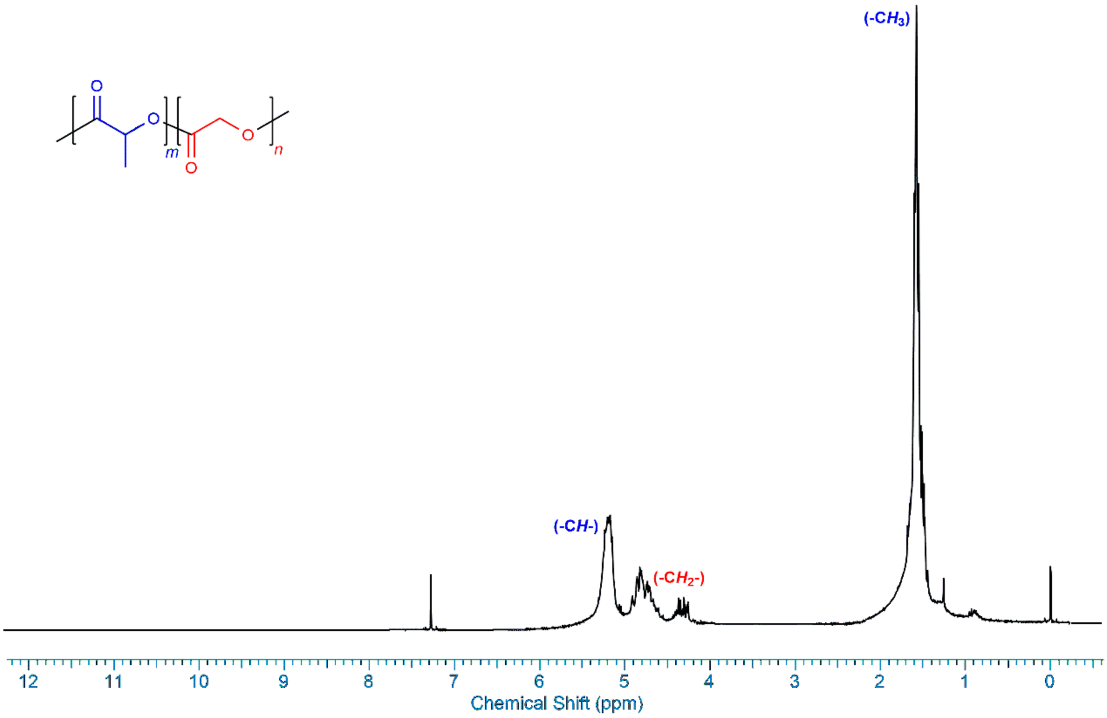

Figure S1. ^{1}H NMR spectrum of PLGA

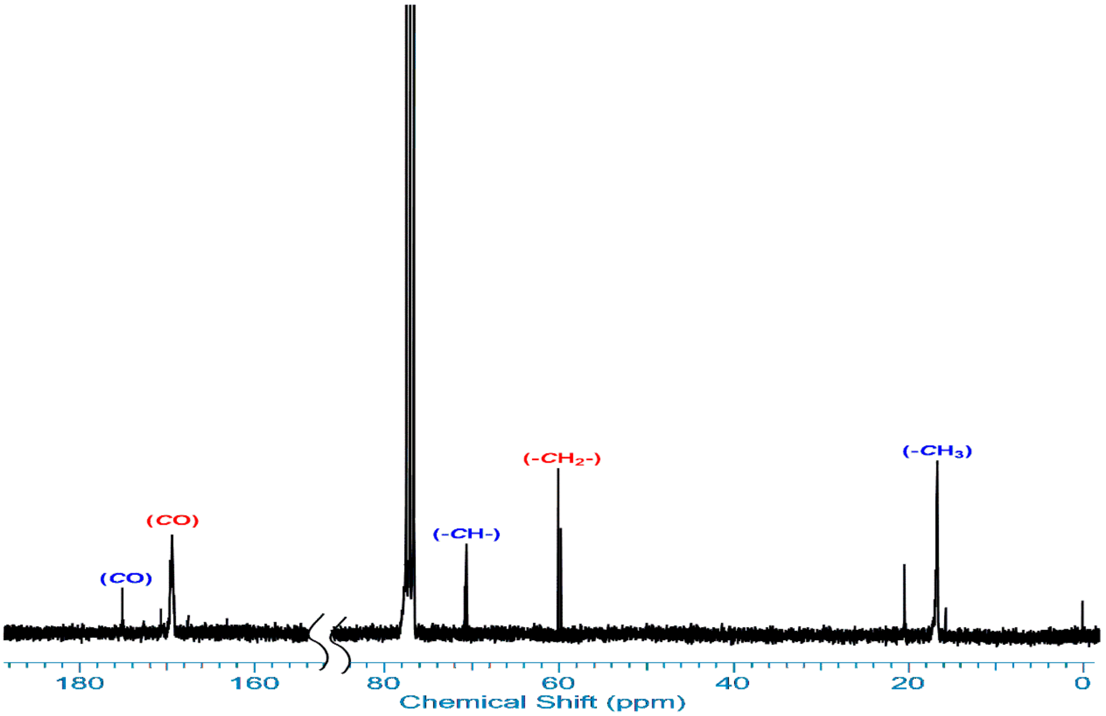

Figure S2. ^{13}C NMR spectrum of PLGA

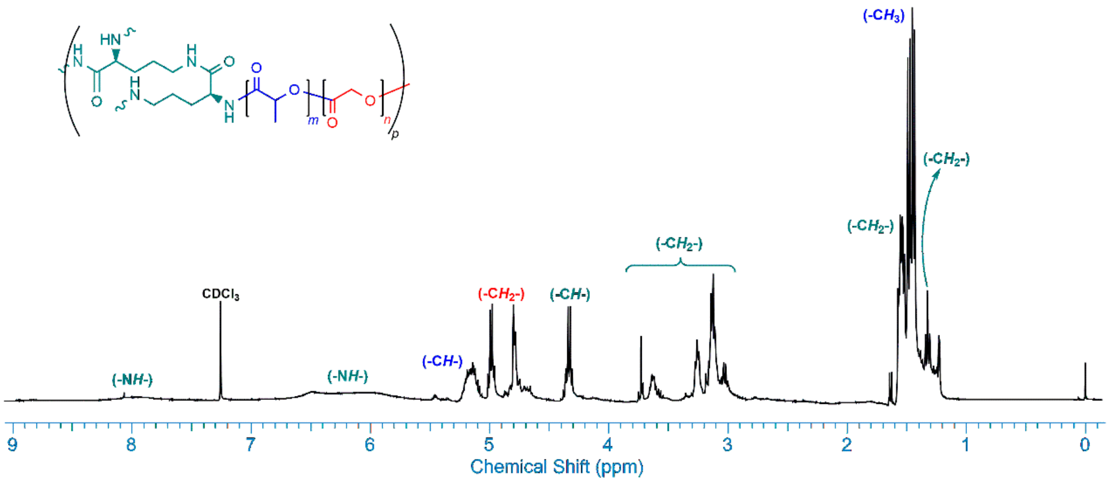

Figure S3. ^{1}H NMR spectrum of PLGA-*L*-Orn

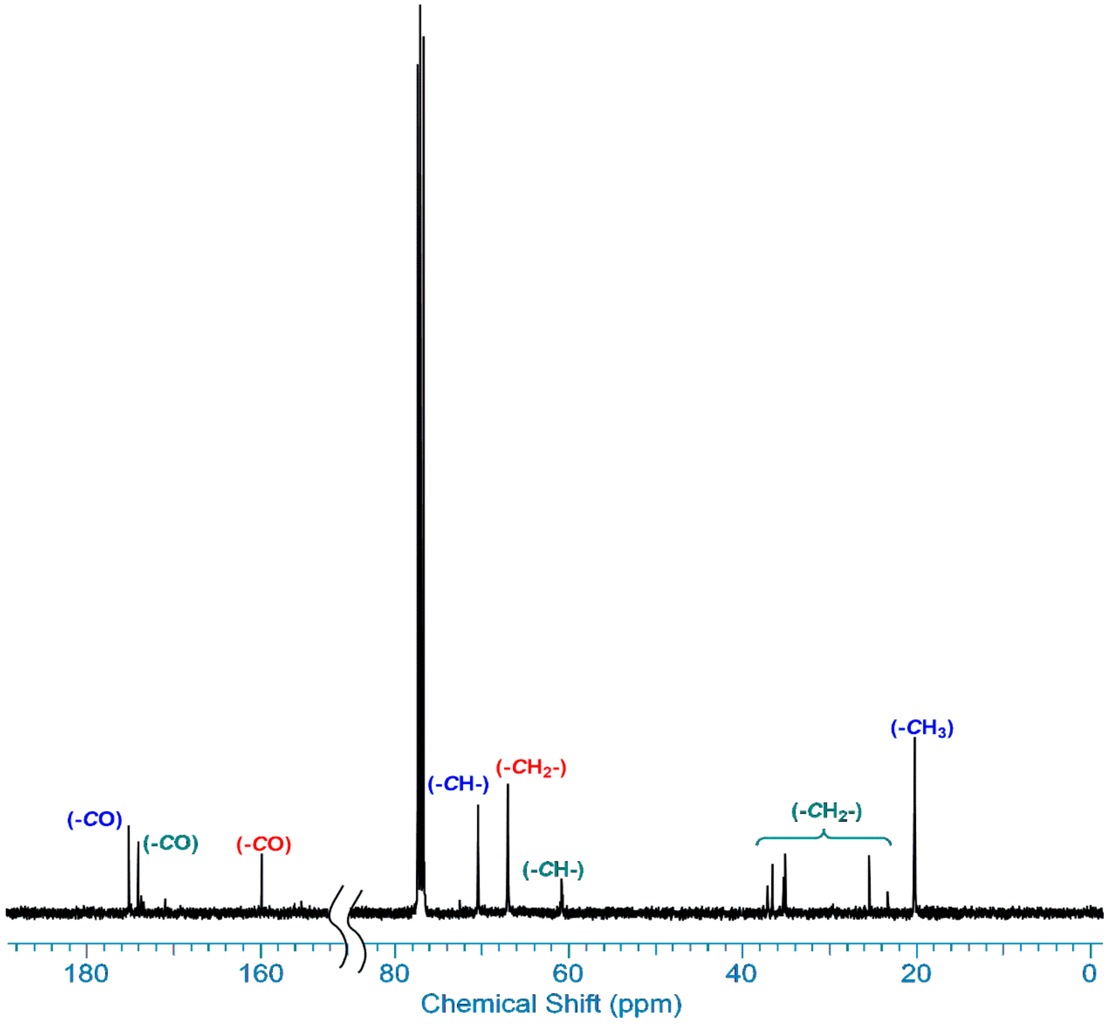

Figure S4. ^{13}C NMR spectrum of PLGA-*L*-Orn

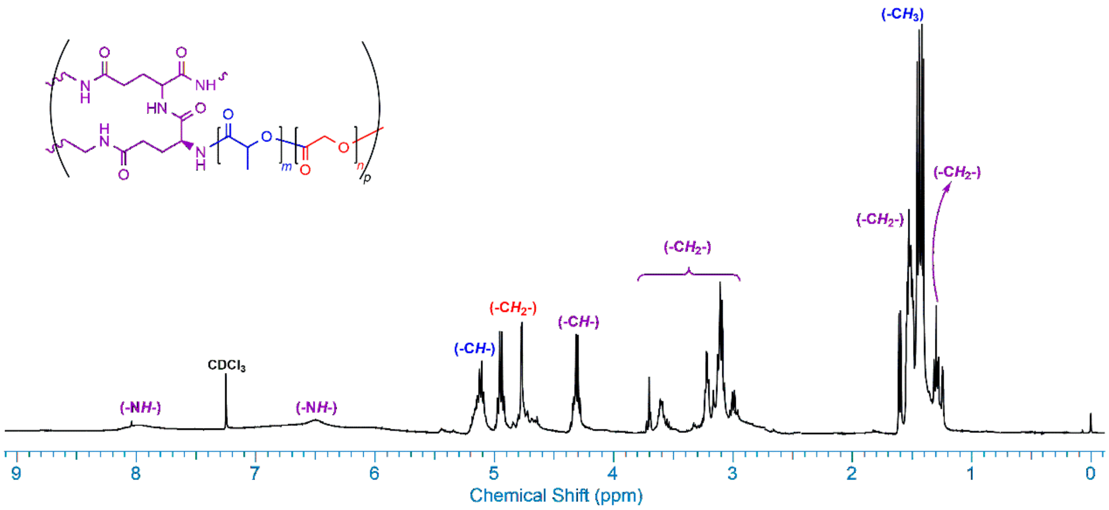

Figure S5. ^{1}H NMR spectrum of PLGA-*L*-Gln

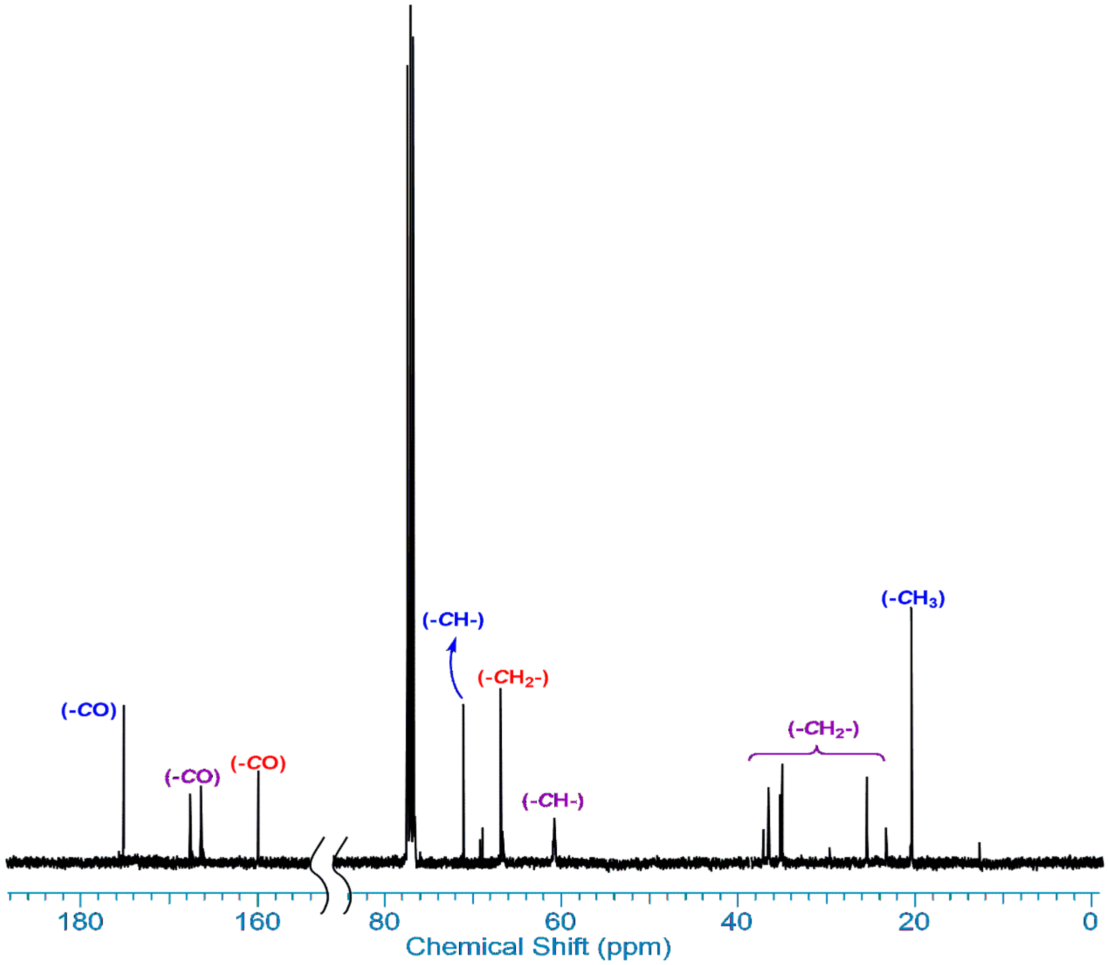

Figure S6. ^{13}C NMR spectrum of PLGA-*L*-Gln

2. FTIR spectrum of PLGA, PLGA-*L*-Orn, PLGA-*L*-Gln

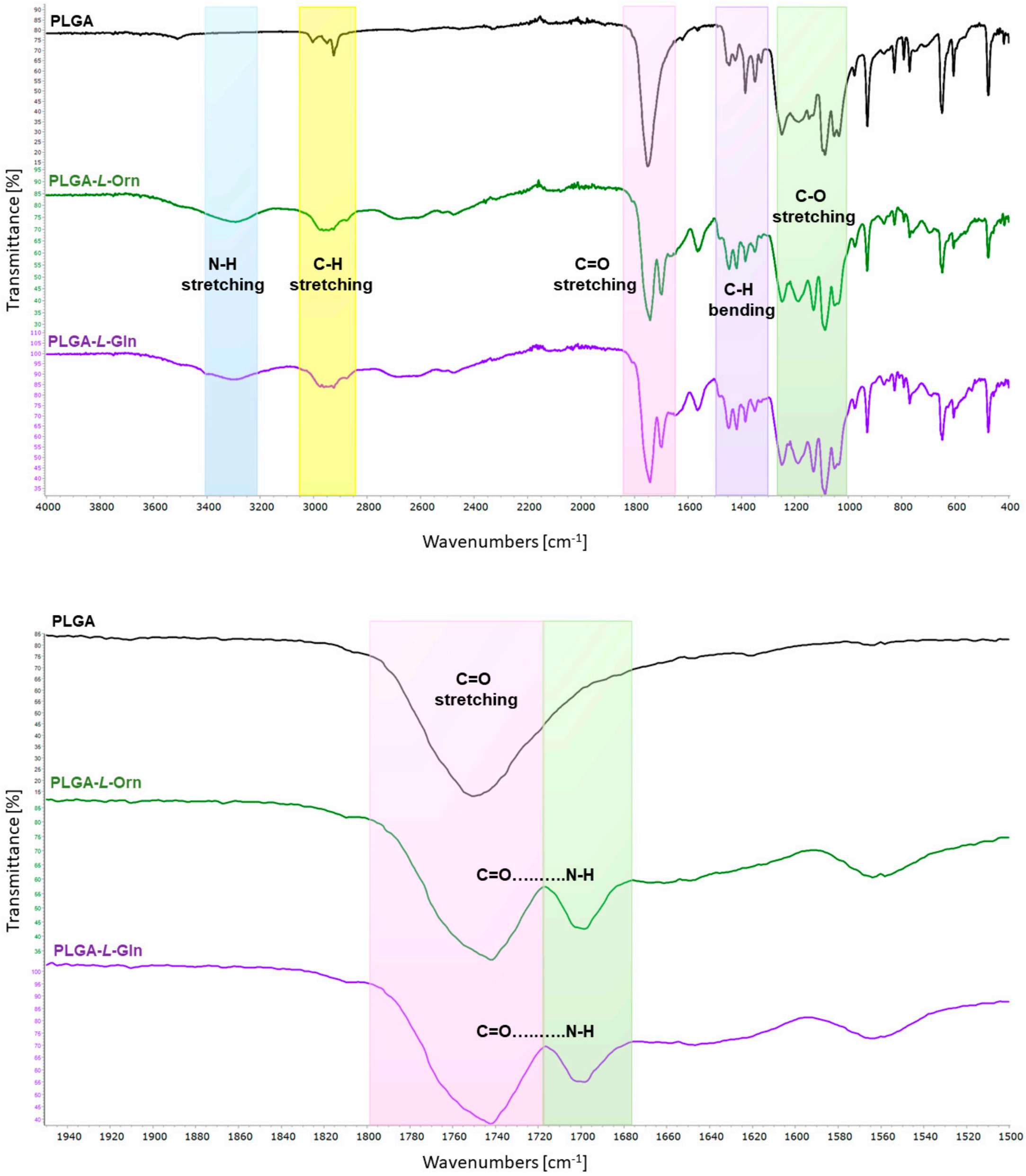

Figure S7. FTIR spectra of PLGA, PLGA-*L*-Orn, PLGA-*L*-Gln

3. TGA and DSC Thermograms of PLGA, PLGA-*L*-Orn, PLGA-*L*-Gln

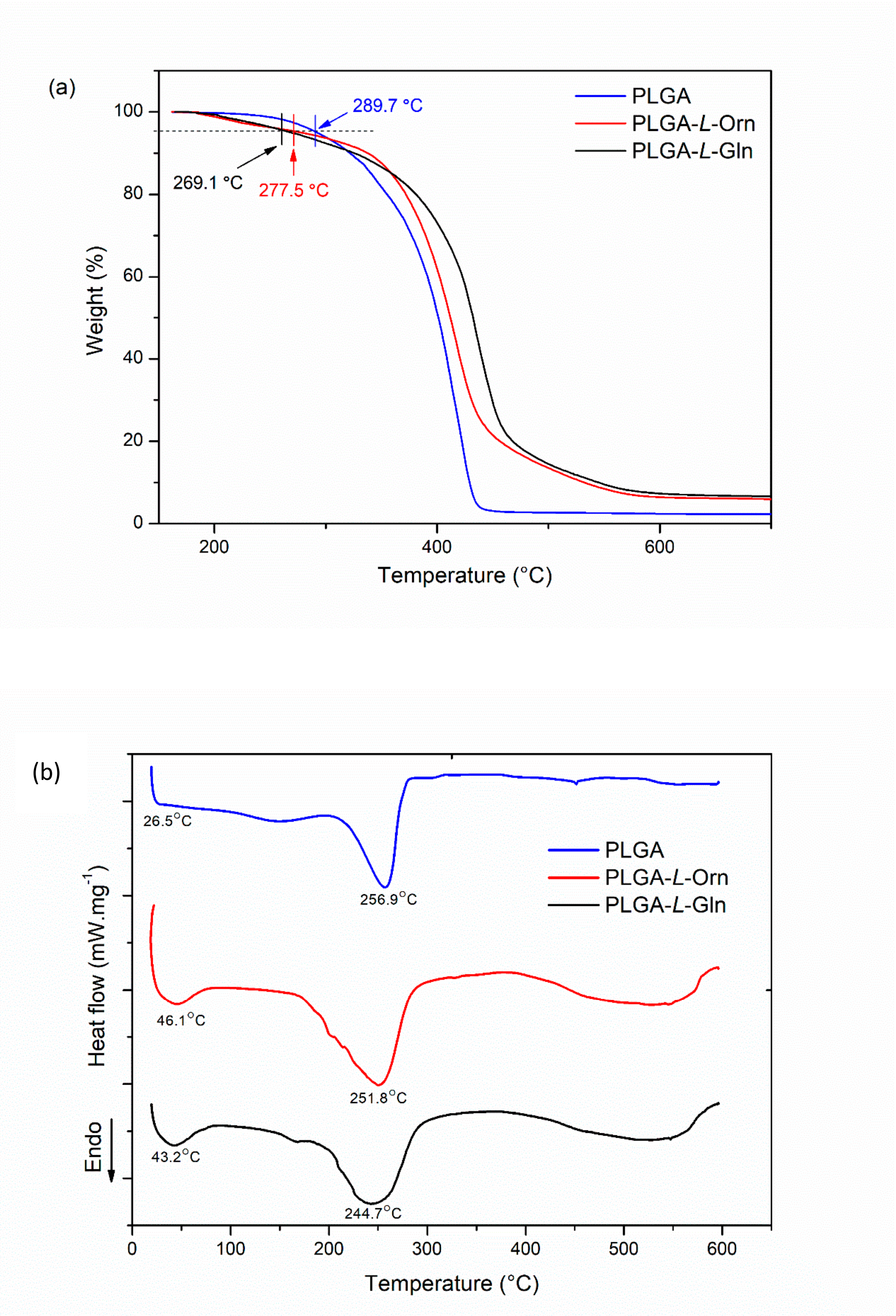

Figure S8. TGA (a) and DSC (b) curves of PLGA, PLGA-*L*-Orn, and PLGA-*L*-Gln

4. Gel permeation chromatography measurement of PLGA, PLGA-*L*-Orn, PLGA-*L*-Gln

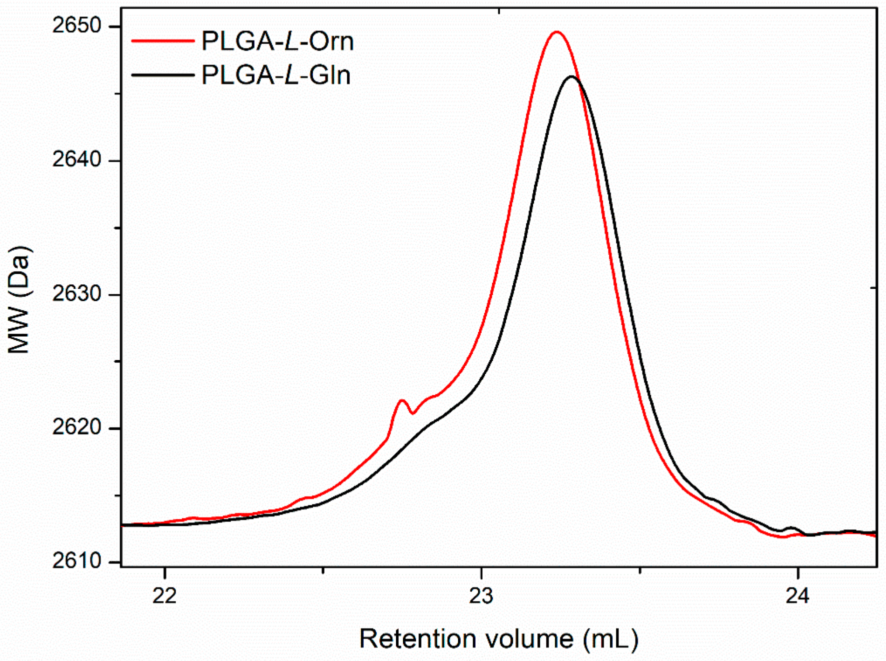

Figure S9. GPC measurement of PLGA-*L*-Orn and PLGA-*L*-Gln
